# LncRNA FLVCR1-AS1 promotes proliferation, migration and activates Wnt/β-catenin pathway through miR-381-3p/CTNNB1 axis in breast cancer

**DOI:** 10.1186/s12935-020-01247-2

**Published:** 2020-06-05

**Authors:** Zhiyu Pan, Junbin Ding, Zhen Yang, Huaqing Li, Hongjian Ding, Qian Chen

**Affiliations:** Department of General Surgery, Minhang Hospital, Fudan University, 170 Xin-Song Road, Shanghai, 201199 China

**Keywords:** FLVCR1-AS1, miR-381-3p, CTNNB1, Breast cancer

## Abstract

**Background:**

Understanding the molecular mechanism of long non-coding RNAs (lncRNAs) in carcinogenesis is conducive for providing potential target for cancers. The role of FLVCR1-AS1 in breast cancer (BC) has not been probed yet.

**Materials and methods:**

qRT-PCR and western blot assays were used to estimate relevant expressions of mRNAs and proteins. CCK8, MTT and EdU were implemented to assess cell proliferation ability. TUNEL was performed to investigate cell apoptosis, whereas transwell assay was performed to test cell migration and invasion capacities. TOP/FOP Flash assay was conducted to determine the activity of Wnt/β-catenin pathway. Luciferase reporter, RNA pull down and RIP assays were performed to verify interaction between genes.

**Results:**

FLVCR1-AS1 was abnormally up-regulated in BC cells. Silencing FLVCR1-AS1 inhibited cell proliferation, migration, invasion, yet accelerating apoptosis. Inhibition of miR-381-3p reversed the tumor restraining impacts of FLVCR1-AS1 depletion on BC progression. Additionally, CTNNB1 was recognized to be targeted by miR-381-3p. FLVCR1-AS1 aggravated BC malignant progression via up-regulation CTNNB1 through sponging miR-381-3p.

**Conclusion:**

FLVCR1-AS1 regulates BC malignant behavior via sequestering miR-381-3p and then freeing CTNNB1, implying a promising target for BC therapy.

## Background

Breast cancer (BC), as one of the most frequently diagnosed cancer types in women, accounts for approximately 29% of all female malignancy cases [1, 2]. Although clinical advances, such as mastectomy, radiotherapy, chemotherapy and even systemic treatment have been achieved in decades, the 5-year overall survival of BC patients is still poorer than anticipation, especially in metastasis cases [3]. Despite an array of gene-expression signatures have been discovered as diagnostic and treatment biomarkers in BC, the molecular function of long non-coding RNAs (lncRNAs) in this field remains on the initial research period. Exploring the mechanism of lncRNA in the pathological processes of BC is of vital importance for future practical target prognosis.

LncRNAs, with over 200 nucleotides in length, belong to a category of RNAs that lack the ability to encode proteins [4]. They have been manifested to participate in various biological activities, such as proliferation, differentiation, transcriptional modification, apoptosis, cell invasion and migration [5–9]. Furthermore, lncRNAs have emerged as novel focuses of clinical applications, since they play pivotal role in human malignancies [10–12].

The prevalent ceRNA mechanism has proofed that lncRNAs can function as competing endogenous RNAs (ceRNAs) for their interaction with sequestered microRNAs (miRNAs), resulting in elevated expression of downstream target genes [13]. In BC, previous studies have found that some lncRNAs were dysregulated and predicted clinical prognosis. For instance, RHPN1-AS1 was found as abnormally up-regulated in BC and facilitated malignant phenotypes in vitro [14]. In addition, Li et al. [15] discovered that lncRNA HOXC13-AS, which was significantly up-regulated in BC, enhanced cell proliferation ability through up-regulating PTEN expression via suppressing miR-497-5p.

Previous studies have revealed the aberrant elevation of FLVCR1-AS1 in several tumors via being engaged in the lncRNA-miRNA-mRNA ceRNA network. For example, in gastric cancer (GC), enriched FLVCR1-AS1 promoted malignant behaviors of GC cells by its ceRNA role of c-Myc through targeting miR-155. Besides, FLVCR1-AS1 overexpression tended to result in poor prognostic outcomes in GC cases [16]. FLVCR1-AS1 contributed to cellular activities in glioma through targeting miR-4731-5p/E2F2 signaling [17]. Also, FLVCR1-AS1 facilitated biological behaviors of ovarian cancer cells via regulating miR-513/YAP1 signaling [18]. FLVCR1-AS1 sponged miR-485-5p to modulate biological behaviors in human cholangiocarcinoma [19]. However, the expression profile, specific function and acting mechanism of FLVCR1-AS1 in BC have not been elucidated yet. Hence, present study aimed to investigate whether and how FLVCR1-AS1 functions in BC.

## Methods

### Cell culture

Human normal breast epithelial cell (MCF-10A) and BC cells (MDA-MB-231, T47D, BT-474, SKBR3, MCF7) were bought from Chinese Academy of Sciences (Beijing, China). Cells were grown in RPMI-1640 medium (Invitrogen, Carlsbad, CA, USA) containing 10% fetal bovine serum (FBS; Invitrogen) and 1% penicillin/streptomycin (Sigma-Aldrich, Milan, Italy) with 5% CO_2_ at 37 °C. LiCL (Taili industrial co. LTD, Shanghai, China), the Wnt/β-catenin pathway activator, was added into culture medium for treating SKBR3 and MCF7 cells.

### Cell transfection

SKBR3 and MCF7 cells were transfected with specific shRNAs against FLVCR1 (sh-FLVCR1-AS1#1#2), MYC (sh-MYC) and negative control (shCtrl), as well as pcDNA3.1/CTNNB1, pcDNA3.1/MYC and the empty pcDNA3.1 vector (all purchased from GenePharma, Shanghai, China), separately. The miR-381-3p mimics, miR-381-3p inhibitor, NC mimics and NC inhibitor were simultaneously constructed by GenePharma. Transfection was conducted for 48 h in light of the protocol of Lipofectamine2000 (Invitrogen)

### qRT-PCR

TRIzol reagent (Takara, Otsu, Japan) was used for extracting total RNA from SKBR3 or MCF7 cells. Subsequently, total RNAs were reversely transcribed into cDNAs under Reverse Transcription Kit (Takara). The qRT-PCR was performed with utilization of SYBR Green real-time PCR Kit (Takara) on the Bio-Rad CFX96 system (Bio-Rad, Hercules, CA). Fold expression changes were calculated via 2^−ΔΔCt^ method, with GAPDH/U6 as reference gene.

### Cell counting kit-8 (CCK-8) assay

Cells (1 × 10^4^) in 96-well plates were cultured with10 µL CCK-8 reagent over specific time points. Absorbance was evaluated at 450 nm using a microplate reader (Bio-Tek Instruments, Hopkinton, MA, USA).

### Colony formation assay

1 × 10^3^ cells were cultured in 6-well plates for 2 weeks. After fixed in methanol (Solarbio, Beijing, China), colonies were processed with crystal violet (Sigma-Aldrich). Visible colonies were counted manually under microscope (Olympus, Tokyo, Japan).

### TUNEL assay

TUNEL staining assay was performed using In Situ Cell Death Detection Kit (Roche, Mannheim, Germany). Following nuclei staining with DAPI (Sigma-Aldrich), relative fluorescence intensity was determined via EVOS FL microscope (Olympus).

### Transwell assay

2 × 10^4^ cells in the top compartment were added with serum-free medium, while medium containing 10% FBS was placed into bottom chamber. After 48 h, migrated cells were fixed with paraformaldehyde (Solarbio) and dyed in crystal violet solution. Invasion assay was performed using the upper chamber was pre-coated with Matrigel (BD Biosciences, Shanghai, China). The number of migrated or invaded cells was captured via a microscope (Olympus).

### Western blot

Total protein was obtained from cells which were lysed by RIPA lysis buffer. Protein concentration was measured with BCA Kit (Pierce, Appleton, WI, USA). We isolated proteins with SDS-PAGE, which were moved to PVDF membranes. 5% skimmed milk was used for blocking membranes, and then were co-cultured with primary antibodies for p53 (ab32389), Bax (ab32503), Bcl-2 (ab185002), MMP2 (ab215986), MMP7 (ab205525), AKT (ab179463), p-AKT (ab38449), β-catenin (ab32572), p65 (ab16502), p-p65 (ab6503) and GAPDH (ab8245) from Abcam (Cambridge, USA). GAPDH was used as internal control. Secondary antibodies were added to incubate for 1 h. Amount of proteins was examined via chemiluminescence detection system. IMAGEJ software (National Institutes of Health, Bethesda, Maryland, USA) was utilized to quantify protein bands, with GAPDH as normalized control.

### Chromatin immunoprecipitation (ChIP)

ChIP experiment was processed with usage of Magna ChIP Kit (Millipore, Darmstadt, Germany). After cross-linked chromatin was sonicated to 200–300-bp fragments by ultrasound, lysates were immunoprecipitated with anti-MYC or anti-IgG. Precipitated chromatin DNA was detected by RT-qPCR.

### Luciferase reporter assay

For FLVCR1-AS1 promoter analysis, the pGL3-Basis reporter (Promega, Madison, WI, USA) vector containing FLVCR1-AS1 promoter was co-transfected with pcDNA3.1/MYC and empty pcDNA3.1 vector, or shCtrl and sh-MYC into cells. The wild-type (WT) and mutant (Mut) binding sites of miR-381-3p mimics in FLVCR1-AS1 sequence or CTNNB1 3′UTR was sub-cloned into pmirGLO dual-luciferase vector (Promega) to construct FLVCR1-AS1-WT/Mut or CTNNB1-WT/Mut, then co-transfected with miR-381-3p mimics or NC mimics into cells. To conduct TOP/FOP-Flash analysis, TOP/FOP-Flash (Genechem) was co-transfected into SKBR3 and MCF7 cells along with sh-FLVCR1-AS1#1 or shCtrl. The luciferase activity was lastly determined via Dual-Luciferase Reporter Assay System (Promega).

### MTT assay

1 × 10^3^ cells in 96-well plates were mixed with 20 µL MTT reagents over 24, 48,72 and 96 h. Later, dimethyl sulfoxide (DMSO) was added. The optical density at 490 nm was analyzed by micro-plate reader (Bio-Tek Instruments, Hopkinton, MA, USA).

### Subcellular fractionation

Subcellular isolation of RNAs in SKBR3 and MCF7 cells was performed by Cytoplasmic and Nuclear RNA Purification Kit (Norgenbiotek Corporation, Thorold, ON, Canada), followed by fraction analysis via qRT-PCR.

### Fluorescence in situ hybridization (FISH) Assay

Fluorescence-conjugated FLVCR1-AS1 probes were produced by Bersinbio Company (Guangzhou, China). BC cells in paraformaldehyde were dehydrated with ethanol. Air-dried cells were denatured for incubation with FISH probes utilizing hybridization reaction buffer overnight. After washing by ×2 saline-sodium citrate, cells were dyed in Hoechst and the results were recorded by Zeiss LSM800 confocal laser microscopy (Zeiss, Oberkochen, Germany).

### RNA pull down assay

The FLVCR1-AS1-WT, FLVCR1-AS1-Mut and NC were biotin labeled into Biotin FLVCR1-AS1 WT, Biotin FLVCR1-AS1 Mut and Biotin Ctrl, severally. Then, cell lysates were cultured with the biotinylated probe and M-280 streptavidin magnetic beads (Sigma-Aldrich). The miR-381-3p levels were analyzed by qRT-PCR.

### RNA immunoprecipitation (RIP)

RNA-binding protein immunoprecipitation kit (Millipore) was applied for performing the RIP assay. SKBR3 and MCF7 cells were lysed with lysis buffer and then incubated with anti-Ago2 and negative control anti-IgG. RNA enrichment was analyzed by qRT-PCR.

### TUNEL assay

The apoptosis of SKBR3 and MCF7 cells were studied via TUNEL Apoptosis Kit (Invitrogen), with employment of DAPI (Koritai Biotechnology, Beijing, China) for dying. Cells were then observed and captured by fluorescence microscopy (Olympus, Tokyo, Japan).

### Tumor growth in nude mice

Male nude mice were obtained commercially from Shi Laike Company (Shanghi, China). Cells transfected with sh-FLVCR1-AS1#1 or shCtrl were injected subcutaneously into mice. Tumor volumes were recorded every 4 day. All mice were sacrificed after 4 weeks, and tumors were removed, weighed. Approval of this animal study was obtained from the Animal Research Ethics Committee of Minhang Hospital, Fudan University.

### Statistical analysis

GraphPad Prism 7.0 software (La Jolla, CA, USA) was applied for statistical analysis. Results were manifested as mean ± SD. The difference of groups was compared via Student’s *t* test or one way ANOVA analysis. P < 0.05 indicated the statistical significance and all experiments were run in no less than triplicate.

## Results

### FLVCR1-AS1 is aberrantly overexpressed in BC cells and silencing FLVCR1-AS1 can dampen the malignant behavior of BC

**Fig. 1:**
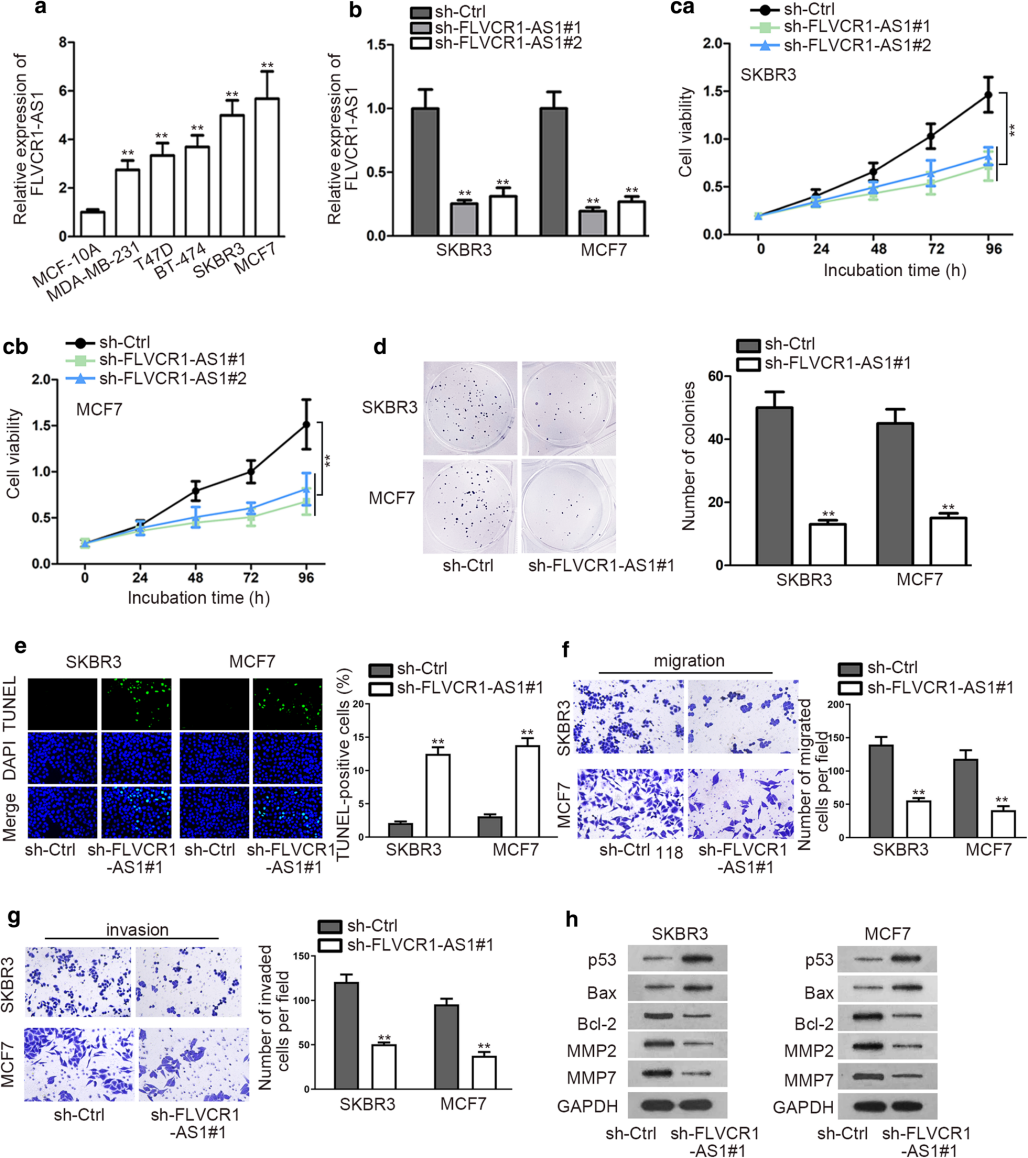
FLVCR1-AS1 is aberrantly up-regulated in BC. **a** qRT- PCR assay was used to detect FLVCR1-AS1 expression in BC cell lines and normal cell line. **b** The relative expression of FLVCR1-AS1 after knocked down was determined by qRT-PCR. **c**, **d** CCK8 and colony formation were performed to assess cell proliferation after silencing FLVCR1-AS1. **e** TUNEL was performed to investigate apoptosis after knockdown of FLVCR1-AS1. **f**, **g** Transwell assays were conducted to determine cell migration and invasion abilities respectively after silencing FLVCR1-AS1. **h** Western blot was performed to examine the expression of proteins associated with cell apoptosis and migration. ^**^P < 0.01

Firstly, qRT-PCR was carried out to explore the expression status of FLVCR1-AS1 in BC cells. As a result, we discovered that FLVCR1-AS1 was significantly up-regulated in BC cell lines (MDA-MB-231, T47D, BT-474, SKBR3 and MCF7) than normal MCF-10A cells (Fig. 1a). Before conducting loss-of-function experiments, we ensured the suppressed expression of FLVCR1-AS1 in SKBR3 and MCF7 cells (Fig. 1b). Thereafter, CCK8 and colony formation assays evaluated that FLVCR1-AS1 inhibition evidently crippled cell vitality (Fig. 1c). After counting colonies, we confirmed that FLVCR1-AS1 depletion could suppress BC cell proliferation (Fig. 1d). Next, TUNEL assay indicated significant increase of TUNEL positive cells after FLVCR1-AS1 knockdown, revealing suppression of FLVCR1-AS1 enhanced apoptosis ability (Fig. 1e). Moreover, we unveiled that compared with control group, knockdown of FLVCR1-AS1 apparently diminished the number of migrated and invaded cells (Fig. 1f, g). These functional experiments revealed that FLVCR1-AS1 overexpression in BC was positively related to BC progression in vitro. Subsequently, western blot manifested that silencing FLVCR1-AS1 notably augmented the expression of p53, Bax, but declined the level of Bcl-2, MMP2 and MMP7 (Fig. 1h). Expression change of these proteins further validated the tumor inhibition effect of FLVCR1-AS1 knockdown.

### MYC enhances the transcriptional activity of FLVCR1-AS1

We supposed that transcription factor might play a role for the abnormal overexpression of FLVCR1-AS1 in BC. We found potential transcription factor responsible for FLVCR1-AS1 regulation via UCSC and Jaspar database (http://jaspar.genereg.net/). We selected MYC among all the transcription factors. Because MYC has been reported to drive signaling pathways and further promotes aggressive BC tumors. Identification of MYC target genes is crucial in signaling pathways that facilitates tumor development [20].

**Fig. 2:**
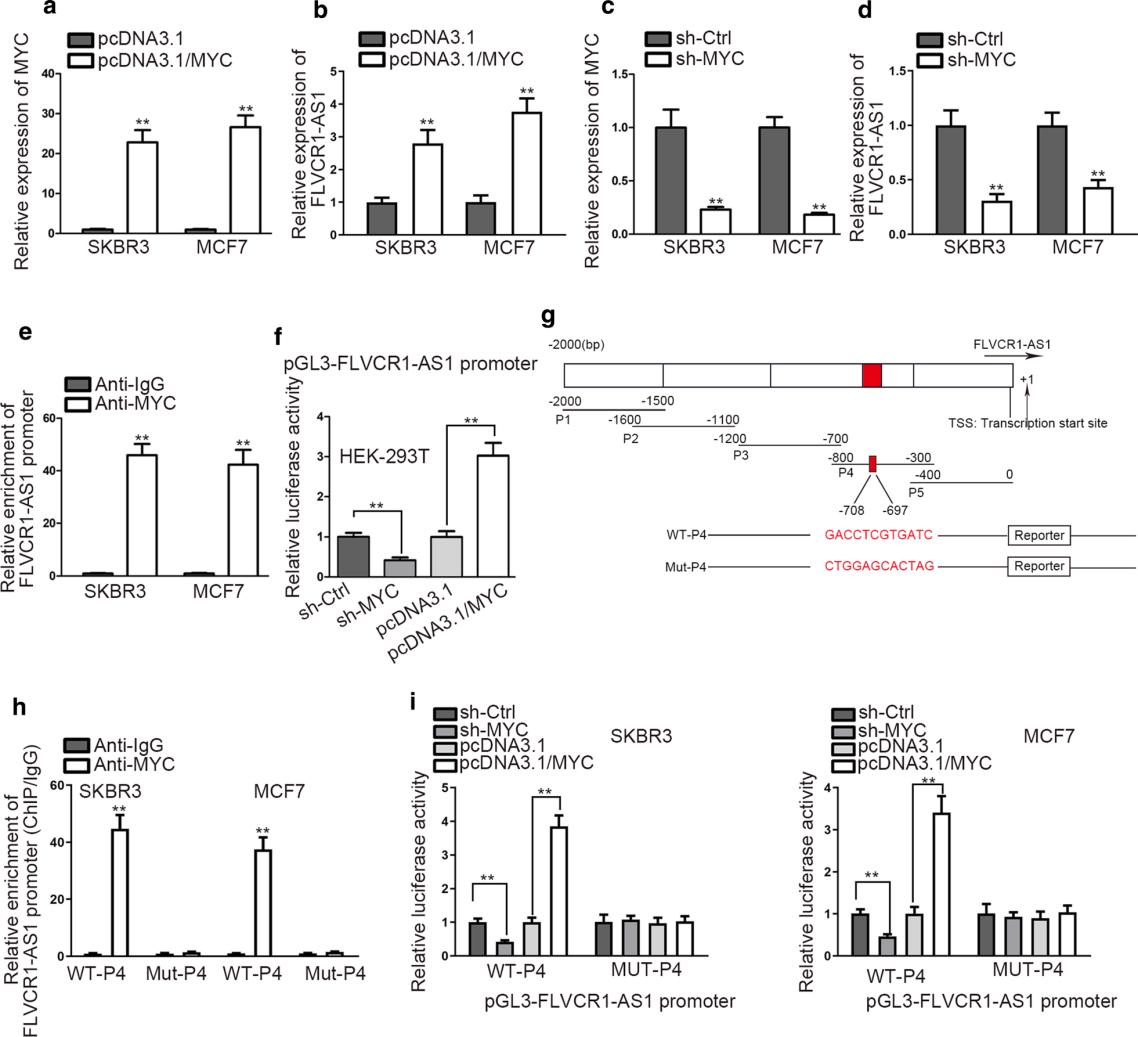
MYC stimulates FLVCR1-AS1 transcription. **a** qRT-PCR analysis was used to determine the expression of MYC after transfecting with pcDNA3.1 and pcDNA3.1/MYC plasmid. **b** qRT-PCR detected the expression of FLVCR1-AS1 after MYC overexpression. **c** qRT-PCR was used to determine expression of MYC after transfecting with sh-Ctrl and sh-MYC. **d** The expression of FLVCR1-AS1 was detected after MYC knockdown. **e** ChIP assay using anti-MYC and anti-IgG was performed to explore the affinity of MYC for FLVCR1-AS1 promoter. **f** HEK-293T was co-transfected with sh-Ctrl, sh-MYC, pcDNA3.1 and pcDNA3.1/MYC vector and a luciferase reporter for 48 h was performed. **g** Potential MYC binding site with FLVCR1-AS1 promoter region was detected on Jaspar. H. ChIP assay was performed to determine the specific sequence in FLVCR1-AS1 promoter for the binding with MYC. **i** Luciferase reporter was conducted to investigate the effect of MYC on FLVCR1-AS1 transcription activity in SKBR3 and MCF7 cells. ^**^P < 0.01

To determine the effects of MYC on FLVCR1-AS1, we overexpressed the expression of MYC (Fig. 2a) and then observed a significant up-regulation of FLVCR1-AS1 in MYC-overexpressed BC cells (Fig. 2b). Conversely, FLVCR1-AS1 expression was significantly decreased after knockdown of MYC (Fig. 2c, d). Above findings suggested the positive influence of MYC in modulating FLVCR1-AS1 expression. Through ChIP assay, we found that FLVCR1-AS1 promoter was largely enriched by antibodies targeting MYC rather than by those against IgG, which suggested that MYC interacted with FLVCR1-AS1 promoter (Fig. 2e). To probe into the impact of MYC on FLVCR1-AS1 transcription, luciferase reporter assay was performed in HEK-293T cell lines. Consequently, we observed that the promoter activity was predominantly attenuated after knockdown of MYC, but enhanced in the presence of MYC overexpression (Fig. 2f). This demonstrated that MYC expedited the transcription of FLVCR1-AS1.

We then detected specific sequence on FLVCR1-AS1 promoter for the binding with MYC by browsing Jaspar. We found that − 697 to − 708 sites were the putative MYC-binding sites at the fragment P4 in FLVCR1-AS1 promoter region (Fig. 2g). Next, we performed ChIP assay to verify this putative MYC binding site. We found that MYC could bind to the P4 fragment in FLVCR1-AS1 promoter region (Fig. 2h). Then, luciferase report was performed to examine the effects of MYC on the transcription of FLVCR1-AS1 in SKBR3 and MCF7 cells. After co-transfection with WT-P4 or MUT-P4, we noticed that MYC overexpression sharply stimulated FLVCR1-AS1 promoter activity, while MYC knockdown greatly suppressed promoter activity in WT-P4 group. However, no significant promoter activity change was observed in MUT-P4 group (Fig. 2i). These data suggested that MYC stimulates FLVCR1-AS1 transcription in BC via interacting with FLVCR1-AS1 promoter at − 697 to − 708 site upstream transcriptional start site (TSS).

### FLVCR1-AS1 contributes to Wnt/β-catenin pathway activation in BC

**Fig. 3:**
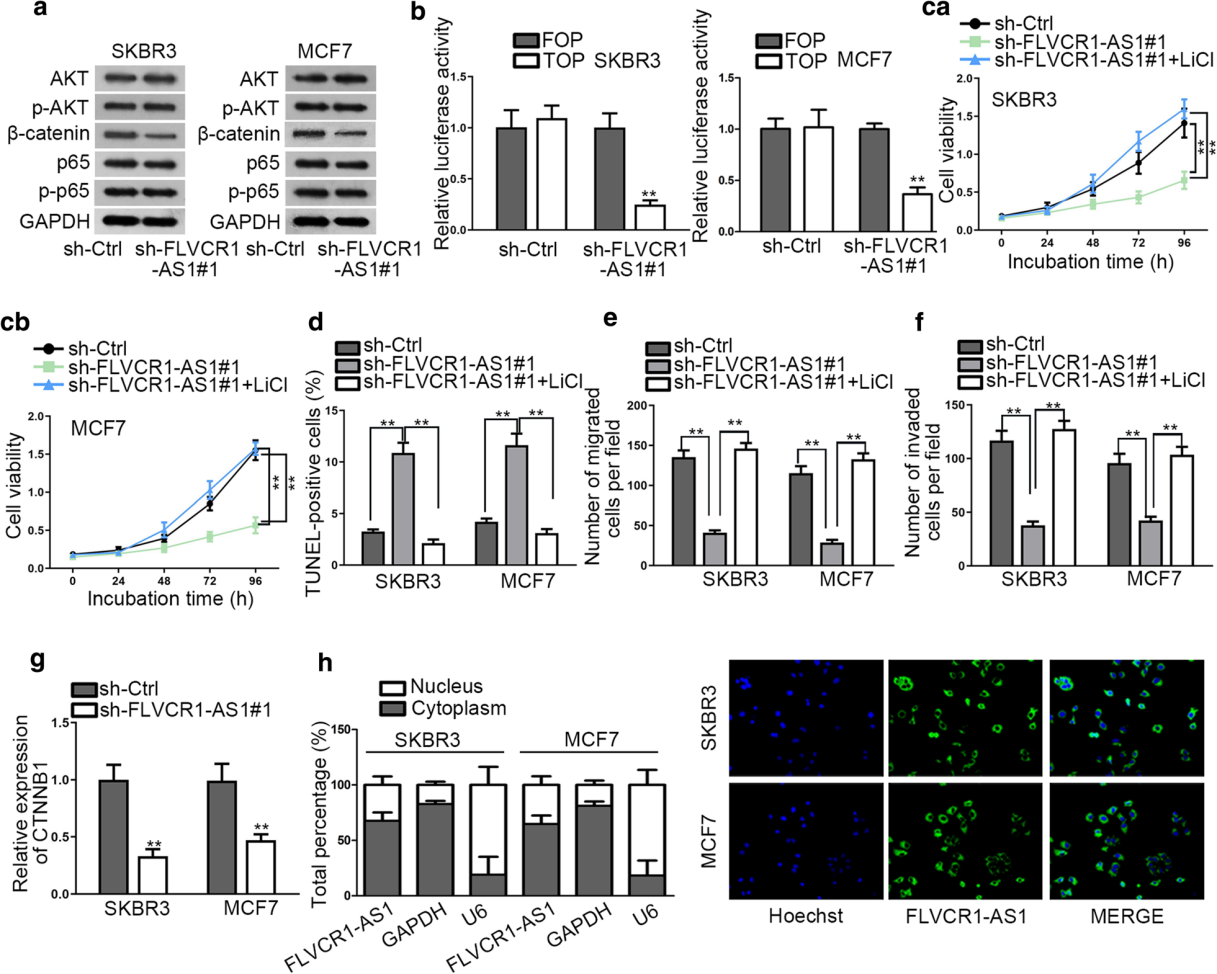
FLVCR1-AS1 activates Wnt/β-catenin pathway. **a** Western blot was performed to measure expression of relevant proteins engaging in common tumor signaling pathway after knockdown of FLVCR1-AS1. **b** FOP/TOP assay was used to verify the activity of Wnt/β-catenin Pathway. **c** MTT was performed to assess cell proliferation after adding agonist of Wnt/β-catenin pathway. **d** TUNEL was performed to investigate apoptosis after adding agonist of Wnt/β-catenin pathway. **e**, **f** Transwell assays were conducted to determine cell migration and invasion abilities respectively after adding agonist of Wnt/β-catenin pathway. **g** qRT-PCR was used to determine expression of CTNNB1 after transfecting with sh-FLVCR1-AS1#1. **h** Subcellular fractionation and FISH were performed to detect the location of FLVCR1-AS1. ^**^P < 0.01

For the sake of understanding the potential mechanism whereby FLVCR1-AS1 affected BC development, we performed western blot to evaluate expression level of proteins related to major pathways associated with BC, such as PI3K/AKT, Wnt and NF-κB pathways. Interestingly, only the level of β-catenin was greatly decreased after FLVCR1-AS1 knockdown instead of p-AKT (PI3K/AKT pathway) or p-p65 (NF-κB) pathway (Fig. 3a). Hence, we preliminarily judged that Wnt/β-catenin signaling pathway might be implicated in FLVCR1-AS1 regulated BC. TOP/FOP Flash assay was performed to evaluate the activity of Wnt/β-catenin after knockdown of FLVCR1-AS1. It manifested that FLVCR1-AS1 knockdown significantly dampened the activity of this pathway (Fig. 3b). To further illustrate whether FLVCR1-AS1 impacted Wnt/β-catenin pathway, we used LiCl, the agonist of Wnt/β-catenin pathway, and evaluated the changes of FLVCR1-AS1 silencing impacted cellular functions. We found that cell proliferation ability was impaired after FLVCR1-AS1inhibition, but recovered in response to LiCl treatment (Fig. 3c). On the contrary, the apoptosis rate was increased after silencing FLVCR1-AS1, but decreased again after adding LiCl (Fig. 3d). Transwell assays found that migrated and invaded cells were decreased after silencing FLVCR1-AS1, but increased sharply again after LiCl treatment (Fig. 3e, f). We then performed qRT-PCR to validate the effects of FLVCR1-AS1 on CTNNB1. Expectedly, FLVCR1-AS1 knockdown led to obvious down-regulation on CTNNB1 expression (Fig. 3g). These findings indicated that FLVCR1-AS1 affected BC malignant behaviors via Wnt/β-catenin pathway. Thereafter, we wanted to know by which manner FLVCR1-AS1 could regulate this pathway. Thus, we detected the subcellular location of FLVCR1-AS1 and determined FLVCR1-AS1 was mainly a cytoplasmic lncRNA with subcellular fractionation and FISH (Fig. 3h), which indicating the possible ceRNA role of FLVCR1-AS1. Hence, we presumed that FLVCR1-AS1 might function as ceRNA to modulate the expression of CTNNB1

### MiR-381-3p is the target of FLVCR1-AS1

**Fig. 4:**
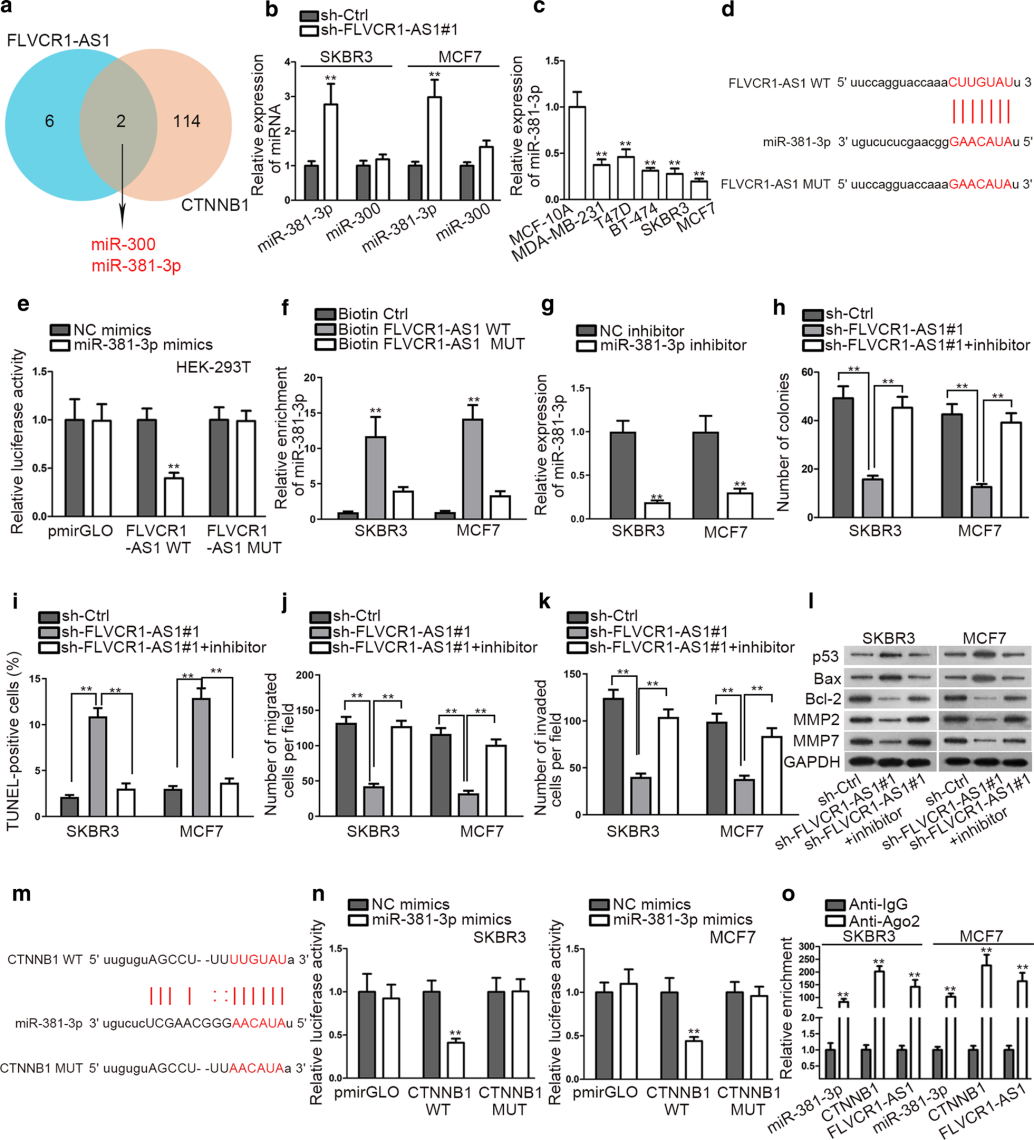
FLVCR1-AS1 competitively binds to miR-381-3p against CTNNB1. **a** Shared combinable miRNAs was detected by Starbase. **b** qRT-PCR analysis was conducted to evaluate miR-381-3p expression after knockdown of FLVCR1-AS1. **c** RT-PCR analysis was conducted to examine the PART1 expression level in BC cell lines and normal cell lines. **d** Putative miR-381-3p binding site with FLVCR1-AS1 predicted on Starbase. **e** Luciferase reporter assay performed in HEK-293T cell line confirmed the interaction between miR-381-3p and FLVCR1-AS1. **f** RNA pull down showed enriched miR-381-3p in biotin-labeled FLVCR1-AS1 wild type group. **g** The transfection efficiency of miR-381-3p inhibitor was assessed. **h** Colony formation was performed to determine cell proliferation among sh-Ctrl, sh-FLVCR1-AS1#1 and sh-FLVCR1-AS1#1 + inhibitor. **i** TUNEL assay was used to examine cell apoptosis among sh-Ctrl, sh-FLVCR1-AS1#1 and sh-FLVCR1-AS1#1 + inhibitor. **j**, **k** Transwell assays were used to measure cell migration and invasion among sh-Ctrl, sh-FLVCR1-AS1#1 and sh-FLVCR1-AS1#1 + inhibitor. **l** Western blot was performed to examine the expression of proteins associated with cell apoptosis and migration. **m** Potential binding site between miR-381-3p and CTNNB1 predicted on Starbase. **n**, **o** Dual luciferase reporter and RIP were performed to verify the interaction. ^**^P < 0.01

We found two shared combinable miRNAs with FLVCR1-AS1 and CTNNB1 by browsing Starbase (Fig. 4a). Of note, the expression of miR-381-3p was enhanced much more dramatically under FLVCR1-AS1 depletion (Fig. 4b). Besides, miR-300 has been reported to be an oncogene in BC [21]. On the contrary, miR-381-3p has been uncovered as tumor inhibitor in BC [22]. Hence, we chose miR-381-3p as the focus of this study. It was disclosed that miR-381-3p was obviously down-regulated in BC cell lines compared with normal one (Fig. 4c). Moreover, putative miR-381-3p binding site to FLVCR1-AS1 was revealed by starBase (Fig. 4d). More importantly, after enhancing miR-381-3p expression, we observed an evident weakness on the luciferase activity of FLVCR1-AS1-WT group, but no distinct luciferase activity change in FLVCR1-AS1-MUT group (Fig. 4e). RNA pull down showed that miR-381-3p expression was more enriched by biotinylated FLVCR1-AS1-WT than negative control groups (Fig. 4f). From above results, we identified that FLVCR1-AS1 served as a miR-381-3p sponge in BC.

In subsequence, rescue assays were implemented to determine the role of FLVCR1-AS1/miR-381-3p axis in BC. Prior to that, we confirmed the inhibition effect of miR-381-3p inhibitor on miR-381-3p expression (Fig. 4g). As anticipated, miR-381-3p inhibition reversed the restrained proliferation in FLVCR1-AS1-silenced BC cells (Fig. 4h). TUNEL manifested that inhibiting miR-381-3p abrogated the pro-apoptosis ability of suppressed FLVCR1-AS1 (Fig. 4i). We also observed that the notably lowered number of migrated and invaded cells upon FLVCR1-AS1 suppression was normalized by miR-381-3p inhibitor (Fig. 4j, k). Then, the results of western blot further proved that inhibition of miR-381-3p offset the anti-tumor effects of FLVCR1-AS1 silence (Fig. 4l).

Potential miR-381-3p binding site inCTNNB1 3′UTR was determined via Starbase (Fig. 4m). Luciferase reporter assay exhibited that enhanced expression of miR-381-3p remarkably attenuated the luciferase activity of CTNNB1-WT (Fig. 4n). RIP found significant enrichment of miR-381-3p, CTNNB1 and FLVCR1-AS1 in RISC (Fig. 4o). These findings indicated that FLVCR1-AS1 competitively bind to miR-381-3p against CTNNB1.

### Upregulated CTNNB1 rescues the suppressive function of depleted FLVCR1-AS1

**Fig. 5:**
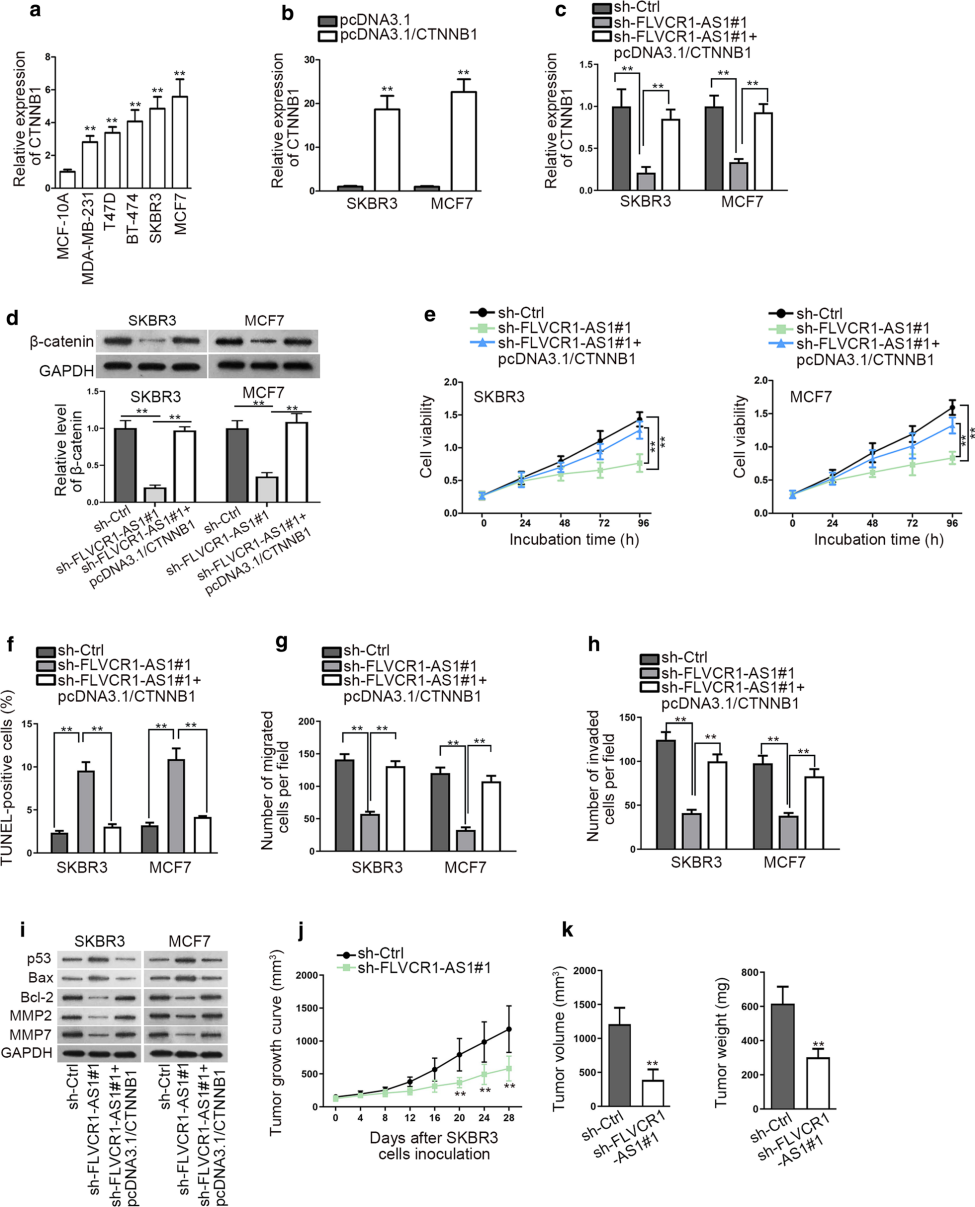
CTNNB1 overexpression rescues the oncogenic function of FLVCR1-AS1. **a** qRT-PCR analysis was conducted to examine the CTNNB1 expression level BC cell lines and normal cell lines. **b** qRT-PCR analysis was used to determine the expression of after CTNNB1 transfecting with pcDNA3.1 and pcDNA3.1/CTNNB1 plasmid. **c**, **d** qRT-PCR and western blot were performed to study the expression of CTNNB1 mRNA and protein. The bands obtained from western blot analysis were further quantified via Image J. **e**–**i** Functional experiments were performed on BC cells in rescue analyses. **j**, **k** Xenograft tumor growth curve, volume and weight between shCtrl and sh-FLVCR1-AS1 mice groups were compared. ^**^P < 0.01

Finally, we intended to validate whether CTNNB1 mediated the function of FLVCR1-AS1 in BC. As observed, CTNNB1 possessed a significant up-regulation in BC cell lines relative to normal MCF-10A cells (Fig. 5a). Then, we verified that co-transfection of pcDNA3.1/CTNNB1 could normalize CTNNB1 level in FLVCR1-AS1-inhibited BC cells (Fig. 5b, c). Result of western blot analysis showed the same effects on β-catenin level (protein form of CTNNB1) (Fig. 5d). Functionally, silencing FLVCR1-AS1 inhibited cell vitality was enhanced again by CTNNB1 overexpression (Fig. 5e). TUNEL result manifested that upregulated CTNNB1 abolished the apoptosis-facilitating effects of FLVCR1-AS1 knockdown (Fig. 5f). Transwell results suggested that overexpressing CTNNB1 sharply reversed the hindered motility caused by depleted FLVCR1-AS1 (Fig. 5g, h). Such observation was further confirmed in Fig. 5i.

Xenografts model was used to further validate the effects of FLVCR1-AS1 on tumor growth in vivo. As indicated, suppression of FLVCR1-AS1 conspicuously slowed down the rate of tumor growth compared to control group (Fig. 5j). We also found apparently lessened volume and weight in tumors with FLVCR1-AS1 inhibition in comparison to those without (Fig. 5k). These experiments in vivo demonstrated that FLVCR1-AS1 promoted BC tumor growth in vivo. Together, FLVCR1-AS1 exerts its oncogenic effects on the malignant behaviors of BC by elevating CTNNB1 level through sponging miR-381-3p.

## Discussion

Despite rapid progression in the diagnosis and treatment and of BC, the mortality rate still remains a challenge that needs the determination of sensitive targets. To improve early diagnosis and therapeutic methods, identifying novel molecular targets for BC is becoming increasingly paramount. Accumulating studies have uncovered the crucial ceRNA roles of lncRNAs in the occurrence and development of a wide array of human diseases [23–26]. Increasing reports have demonstrated that lncRNAs can play important regulatory role in many biological activities and are correlated with the carcinogenesis of cancers [25]. Determining the relationship between lncRNAs and their downstream targets would contribute to the diagnosis and treatment of patients with BC.

In present study, we found that FLVCR1-AS1 was significantly up-regulated in BC cell lines. Knockdown of FLVCR1-AS1 sharply suppressed BC cell proliferation, migration and invasion, while stimulating cell apoptosis in vitro. Besides, the expression of FLVCR1-AS1 was found to be positively correlated with tumor growth, size and volume in vivo, which supported that FLVCR1-AS1 played an oncogenic role in BC. Besides, MYC transcriptionally activated FLVCR1-AS1 in BC.

MiR-381-3p was identified as a potential target gene of FLVCR1-AS1. MiR-381-3p has been discovered to be a tumor suppressor gene and reported to be down-regulated in various human malignancies, including cervical cancer, bladder cancer, oral squamous cell carcinoma as well as BC [27–29]. We verified the interaction between miR-381-3p and cytoplasmic FLVCR1-AS1. Furthermore, miR-381-3p inhibition reversed the suppressing effects of sh-FLVCR1-AS1 on malignant behaviors of BC cells in vitro, which indicated that FLVCR1-AS1 exerted its oncogenic role in BC via sponging miR-381-3p.

We observed that only β-catenin associated with Wnt/β-catenin was significantly down-regulated after FLVCR1-AS1 knockdown. We observed that LiCl, as agonist of Wnt/β-catenin pathway, could abolish the anti-tumor effects of FLVCR1-AS1 knockdown. Subsequently, we identified that CTNNB1 was the target gene of miR-381-3p. Additionally, CTNNB1 has been extensively reported to play an oncogenic role and predicted poor prognosis in multiple cancers. In present study, we found that overexpression of CTNNB1 could abrogate the tumor-inhibiting ability of sh-FLVCR1-AS1. In other words, CTNNB1 up-regulation could rescue the anti-oncogenic function of FLVCR1-AS1 depletion. Together, our finding initially suggested that lncRNA FLVCR1-AS1 could function as miR-381-3p sponge and up-regulate the expression of CTNNB1 and activate Wnt/β-catenin pathway, consequently aggravating BC malignant progresses.

## Conclusion

This study found the MYC/FLVCR1-AS1/miR-381-3p/CTNNB1 axis in BC initially. We identified the oncogenic role of FLVCR1-AS1 in BC progression. It could hopefully be applied as a potential diagnostic and therapeutic biomarker for patients with BC.

## Data Availability

Research Data are not shared.

## Abbreviations

lncRNAs: Long non-coding RNAs
BC: Breast cancer
ceRNAs: Competing endogenous RNAs
miRNAs: MicroRNAs
EMT: Epithelial-to-mesenchymal transition
GC: Gastric cancer
CCK-8: Cell Counting Kit-8
ChIP: Chromatin Immunoprecipitation
FISH: Fluorescence in situ hybridization assay
RIP: RNA Immunoprecipitation
